# Enzyme Immobilization on Gold Nanoparticles for Electrochemical Glucose Biosensors

**DOI:** 10.3390/nano11051156

**Published:** 2021-04-28

**Authors:** Wiktoria Lipińska, Katarzyna Grochowska, Katarzyna Siuzdak

**Affiliations:** Centre for Plasma and Laser Engineering, The Szewalski Institute of Fluid-Flow Machinery, Polish Academy of Sciences, Fiszera 14 St., 80-231 Gdańsk, Poland; wlipinska@imp.gda.pl (W.L.); kgrochowska@imp.gda.pl (K.G.)

**Keywords:** enzyme immobilization, covalent bonding, adsorption, cross-linking, entrapment, self-assembled monolayers, gold nanoparticles, biosensor

## Abstract

More than 50 years have passed since Clark and Lyon developed the concept of glucose biosensors. Extensive research about biosensors has been carried out up to this day, and an exponential trend in this topic can be observed. The scope of this review is to present various enzyme immobilization methods on gold nanoparticles used for glucose sensing over the past five years. This work covers covalent bonding, adsorption, cross-linking, entrapment, and self-assembled monolayer methods. The experimental approach of each modification as well as further results are described. Designated values of sensitivity, the limit of detection, and linear range are used for the comparison of immobilization techniques.

## 1. Introduction

**Figure 1:**
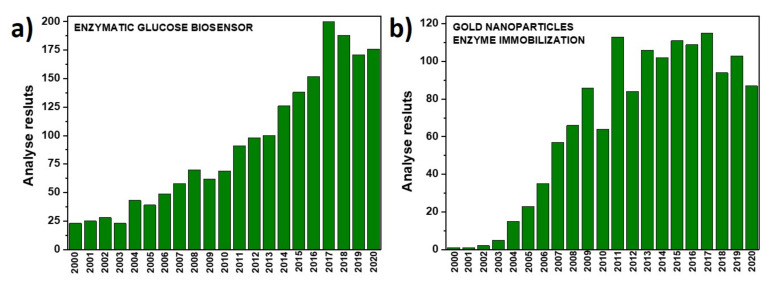
The bar chart showing the number of analyzed results in Web of Science (WoS) for (**a**) enzymatic glucose biosensor, (**b**) gold nanoparticles enzyme immobilization.

The aim of the current review is to provide current knowledge about glucose oxidase immobilization methods on gold nanoparticles for glucose detection. The motivation behind this review comes from the large number of people struggling with diabetes and its complications. It is estimated that over 420 million people suffer from this illness at this moment in time [1]. Therefore, the glucose biosensor market is constantly growing. Nevertheless, there is still a need for improvements in the stability, simplicity and miniaturization of sensors [2]. Moreover, the most popular method to monitor glucose levels in the human body is a single-use strip readout using a portable blood glucose sensor. Unfortunately, this sensor cannot track glucose levels during sleep and when playing sports and causes disruption of the human skin. It should be also underlined that the diabetic must always remember to keep the sensor with them. Therefore, a great deal of effort is made to develop continuous glucose monitoring devices in physiological fluids, especially non-invasive ones. The greatest attention has been focused on electrochemical enzymatic sensors due to selectivity, simplicity of design and high performance [3]. The analysis of research papers where keywords such as “enzymatic glucose biosensor” (Figure 1a) or “gold nanoparticles enzyme immobilization” (Figure 1b) were used indicates the strong exponential trend in this topic up to 2017 or 2011, respectively. Therefore, the scientific world recognizes this problem and has made a great effort to find the most suitable and convenient solution.

Electrochemical enzyme-based biosensors are analytical devices composed of a bio-recognition material and a transducer [4]. The working electrode can be modified with, for example, Au nanoparticles (NPs) before enzyme immobilization, or AuNPs can be deposited together with molecules. Enzymes working in the frame of the key and lock analogy can selectively recognize analyte and induce a redox reaction that is finally responsible for the generation of an electric signal. The electric signal correlates to the substrate concentration, but this relationship is strongly affected by the sensor construction and the environment of the measurement. The electrochemical biosensor can be evaluated based on its linear range, the limit of detection (LOD) and sensitivity, recognized as three major parameters describing sensing device characteristics [5]. Electrochemical techniques used for glucose detection can usually be divided into three categories: potential, current and impedance-based methods. At the same time, the enzymatic reaction which is monitored during measurement can cause charge accumulation or a potential drop/increase (potentiometric), charge generation (amperometric) or changes in resistance (impedimetric) [5]. In addition, the electrochemical biosensors can be divided into first-, second-, and third-generation sensors depending on the type of mechanism occurring during the period when the glucose molecule interacts with the sensing electrode [6]. In order to prepare the perfect biosensor, characterized by a low limit of detection, wide linear range, high sensitivity, good selectivity and the absence of non-specific bindings, effective electron transfer between the enzyme and transducer, as well as high stability and an excellent understanding of the interactions between the enzyme modification components are required. The immobilization of an enzyme can have various effects on enzyme activity [7]. It is valuable because this makes it possible to reuse an enzyme multiple times, thereby extending its life span as well as reducing its degradation [8]. In addition, this immobilization influences the improvement of its pH and temperature stability [9]. The selectivity, specificity and activity can be improved after immobilization by changing the conformation of the enzyme [10]. Another benefit of enzyme immobilization is the ability to catalyze reactions in non-aqueous environments. The homogenous product instead of a mixture of enantiomers and isomers can also be obtained [11]. This immobilization increases operational stability [12]. Suitable separation of the enzyme from the platform, medium and product allows the reduction of costs [13]. It should also be emphasized that biocatalytic processes involving enzymes are environmentally friendly [14]. However, enzyme distortion due to the multi-interactions between the support and molecules can possibly occur. Consequently, the enzyme can change properties or even lose its activity. Other examples are the blockage of active centers coming as a result of the unsuitable enzyme orientation and the diffusion limitations [15]. The selection of an appropriate immobilization method for the specific substrate can help prevent these problems.

Gold nanoparticles provide an excellent platform for solving health problems in cancer therapy as well as for chemical and biological sensing [16]. The properties of AuNPs can be tuned by changing their shape, size, and aggregation. The modification of the nanoparticle surface with various types of molecules provides unique properties for further applications, among others, in drugs, nucleic acids, proteins, bacteria and virus detection. Currently, many nanoparticles include the noble metals Au, Ag, Pt, Pd [17,18,19], and oxides (CuO, Cu_2_O, NiO, Fe_2_O_3_ [20,21,22]), as well as bimetallic systems (Au-Pt, Au-Pd, and Cu-Ag [23,24,25]), have been used for glucose detection. Nevertheless, the major benefit of using gold is a higher glucose oxidation current than for other noble metals [26]. Transition metals and their oxides, such as Cu and Ni, have been extensively examined because of their good catalytic performance. However, in the case of Cu and its oxides, high background current and the competitive oxygen evolution reaction can disturb glucose oxidation [26].

The majority of review papers are dedicated to enzyme-based electrochemical sensors and are mainly focused on their further on-body application presenting an attractive external appearance. On the contrary, herein we collected the achievements regarding the immobilization of glucose oxidase onto the gold nanoparticles, as one of the most important steps during sensor fabrication. This unique approach results also from our own experimental route [27,28] enabling us to optimize the procedure toward the most sensitive electrode material. Since glucose oxidase and gold nanostructures are the most applied enzyme and host substrate, respectively, we focused on one type of enzyme and the detected analyte. In this review, the various immobilization methods together with their advantages and disadvantages were thoroughly described and the performances of the final electrode materials were compared.

## 2. Preparation of Gold Nanoparticles

Regarding the sensing platforms where the enzyme plays an important role as a selective recognizer of a particular analyte molecule, the whole fabrication process covers not only enzyme immobilization but also preparation of the conducting platform that enables charge collection and, in consequence, electrochemical detection. Among many nanostructures used as a host for the further anchoring of enzyme molecules are gold nanostructures that are chosen to be used in electrochemical sensors for glucose detection, as they exhibit high biocompatibility and chemical stability [29]. The fabrication of gold nanoparticles, as in the case of all nanomaterials, can be divided into two major approaches: bottom-up and top-down. The synthesis of Au nanoparticles using the bottom-up technique is based on the formation of NPs through the connection of atoms, molecules or smaller particles. According to the top-down route, the material is reduced to smaller parts called nano-sized particles [30]. One of the examples of bottom-up technique is synthesis performed in a liquid environment, such as chemical reduction [31] or the sol-gel synthesis. Such colloidal AuNPs are one of the most used nanomaterials for biosensors. The preparation of AuNPs via chemical reduction consists of two steps where the following compounds are used: (1) reduction ones such as borohydrides, citric and oxalic acid, hydrogen peroxide, formaldehyde, acetylene and others which should provide an electron for the reduction in Au^3+^ and Au^+^ ions in order to form a Au^0^ colloid; (2) NPs stabilizers like polymers, trisodium citrate dihydrate, sulfur and phosphorus ligands, dendrimers and surfactants [32]. The main advantage of this method is simplicity; however, some reduction compounds can be toxic [33]. Another example of the bottom-up technique is chemical vapor deposition (CVD) [34]. This technique is based on the condensation of a compound from the gas phase, resulting in the formation of solid material on the substrate [35]. For example, acting according to Chew et al. [36], tin oxide films with AuNPs were fabricated using monobutyl tin trichloride and auric acid precursor solution by AACVD (aerosol-assisted chemical vapor deposition). Top-down techniques include, among others, the magnetron sputtering technique [37] and laser ablation synthesis [38]. Sputtering is a physical vapor deposition process (PVD) based on the interaction of a high purity metal target with an Ar ion plasma and further deposition of metal onto a supporting material [39]. Acting according to K. Grochowska et al. [29] the AuNPs on ITO (indium thin oxide) substrate can be formed by pulsed laser dewetting of sputtered thin gold film. Gold nanoparticles can be also fabricated by the thermal treatment of thin Au layers in a typical electric furnace [40]. There are many other methods of gold nanoparticle preparation, however, those mentioned above are the most commonly used. However, it should be highlighted that if the Au nanoparticles are synthesized as a stable colloid, an additional step should be applied toward the electrochemical sensor fabrication, namely, its permanent anchoring on the conductive substrate. This stage can be realized via spin-coating, printing, selective electrospraying, electrochemical deposition, electroless plating [41,42] and other techniques. The immobilization process also requires much attention, because the conducting support should be uniformly decorated by NPs without any agglomerates that can affect further enzyme immobilization, and hence the performance of the sensor. Therefore, the most convenient way is to couple both processes, AuNP formation and their immobilization, onto the stable and conductive substrate, in one route. Such a strategy eliminates optimization of the additional step and enables further facile up-scaling. The comparison of non-enzymatic electrodes containing AuNPs for glucose detection is shown in Table 1.

As far as non-enzymatic glucose biosensors are concerned, in most cases, the measurements are carried out in alkaline solutions. The sodium or potassium hydroxide solutions have no practical application due to the very high pH which is not found in physiological human body fluids. Therefore, such a glucose sensor can be applied only in very narrow and specific conditions, or after special treatment of the biological samples. If the sensor is predicted for practical use, tests are performed in neutral environments such as phosphate buffer solution in which the pH is similar to that of human body fluids.

## 3. Types of Immobilization Methods

**Figure 2:**
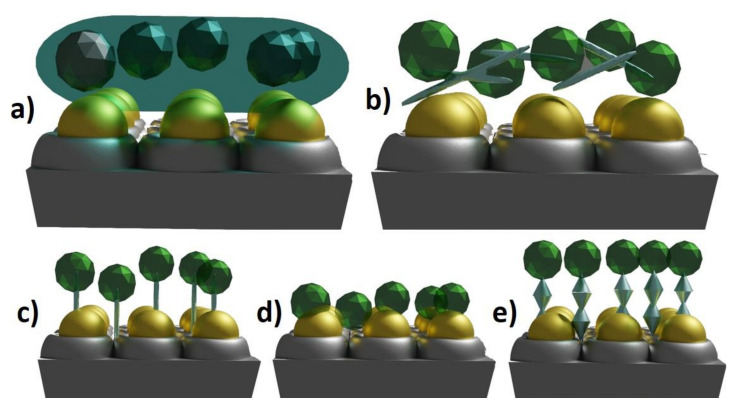
Types of immobilization methods: (**a**) entrapment, (**b**) cross-linking, (**c**) covalent bonding, (**d**) adsorption, (**e**) self-assembled monolayers.

Enzymes have unique catalytic activity as well as excellent selectivity, which results in wide usage in the industrial and medical sectors. However, it should be also kept in mind that enzymes are unstable molecules and can be characterized by high production and separation costs [52]. Enzyme immobilization has received great attention since it can eliminate disadvantages and even widen the range of possible applications. The main advantages of immobilization are multiple or repeatable usage of molecules and improving their stability [53]. There are five most commonly applied methods: covalent bonding, adsorption, cross-linking, entrapment and self-assembled monolayers. The schematic representation of each method is shown in Figure 2.

### 3.1. Covalent Bonding

The method based on covalent bonding involves the creation of the chemical bond known as sharing of electron pairs between the enzyme and the platform on which the molecule is immobilized [54]. The mechanism of this process can be divided into two stages. Firstly, the activation of the substrate using linker molecules, such as carbodiimide or thiol, occurs. Secondly, the other end of the linker molecule is connected with the enzyme [52]. Immobilization by covalent bonding ensures high interaction strength as well as a multipoint attachment. The main disadvantages of this method are complicated procedures such as an unstable linker and costly, toxic reagents [55].

Covalent enzyme immobilization on graphene-gold nanoparticles (GNP) modified interdigitated (di)electrodes (IDE) was investigated by Ge et al. [56]. Firstly, 5 mM of the 16-MUA (16-mercaptoundecanoic acid) was mixed with gold nanoparticles to make a link by –SH and –COOH groups. Then, 200 mM of the EDC (N-ethyl-N′-(3-dimethyl aminopropyl)carbodiimide hydrochloride) and 50 mM of the NHS (N-hydroxysuccinimide) at 1:1 ratio were added for the activation of unbonded –COOH groups and to immobilize –NH_2_ groups from GOx (glucose oxidase). The glucose detection on the graphene-GOx-GNP electrode was compared with the one on the graphene-GOx electrode. As has been already mentioned, the enzyme immobilization for the sample which contains gold was carried out using the covalent bonding method. However, in the case of the graphene-GOx electrode, immobilization via the adsorption method was performed. The glucose sensor containing gold nanoparticles was characterized by greater current changes—caused by glucose addition to the electrolyte—in comparison to the sensor without GNP. The limit of detection for a graphene-GOx electrode was found to be 0.06 mg/mL, whereas for a graphene-GOx-GNP electrode it was reduced to 0.03 mg/mL. Therefore, the addition of gold nanoparticles to the enzyme sensor decreases the LOD. In addition, for the electrode with GNP, the slope was equal to 0.69 µA mg mL^−1^, which is two times higher value than for the sample without gold nanoparticles.

**Figure 3:**
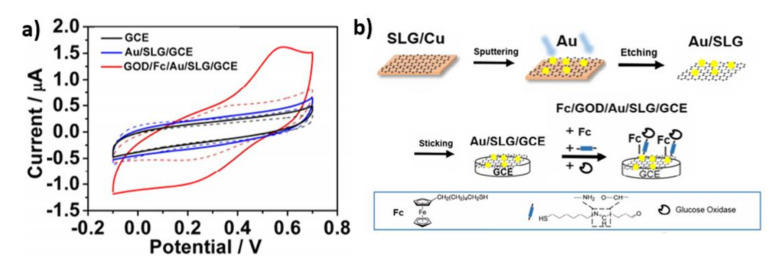
(**a**) CV curves registered for GCE, Au/SLG/GCE, GOD/Fc/Au/SLG/GCE electrodes in PBS solution without (dash line) and with (solid line) 50 µM glucose; (**b**) schematic illustration of fabrication of GOD/Fc/Au/SLG/GCE electrode. Reprinted with permission from [57]; Copyright 2019, Elsevier.

The Au/SLG/GCE (gold nanoparticles on a single-layer graphene glass carbon electrode) was a platform for GOD (glucose oxidase) immobilization using covalently bound –SH groups with gold (Figure 3b) [57]. The gold nanoparticles were used to join the 6-(ferrocenyl)hexanethiol and the 6-amino-1-hexanethiol hydrochloride with the electrode by forming Au-S bonds. Then the aldehyde groups (–CHO)—included in glutaraldehyde—were connected to amino groups (–NH_2_) of the glucose oxidase enzyme and the 6-amino-1-hexanethiol hydrochloride. The 6-(ferrocenyl)hexanethiol (Fc) plays the role of electron transfer medium between the electrode surface and the redox enzyme center. The electrochemical activity for the GCE, the Au/SLG/GCE and the GOD/Fc/Au/SLG/GCE electrodes toward glucose in phosphate buffer solution is shown in Figure 3a. For the GCE and the Au/SLG/GCE samples, any redox peaks in the potential range from −0.1 V to +0.7 V after glucose injection to the solution were not observed. However, the GOD/Fc/Au/SLG/GCE anodic peak at +0.55 V can be interpreted as the glucose oxidation reaction. The detection of ultra-low glucose concentration on the electrode containing Fc and GOD was also confirmed. The limit of detection of the electrode is equal to 0.10 nM.

**Figure 4:**
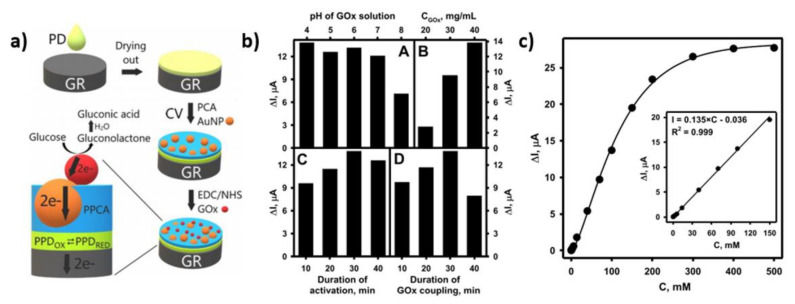
(**a**) Schematic representation of the preparation of a GR/PPD/(AuNP)PPCA-GOx electrode, (**b**) dependence of current changes caused by (**A**) pH changes, (**B**) GOx concentration, (**C**) duration of activation, (**D**) duration of GOx coupling; (**c**) the relationship between current and glucose concentration. Reprinted with permission from [58]; Copyright 2020, Elsevier.

Acting according to Kausaite-Minkstimiene et al., the GR/PPD/(AuNP)PPCA (graphene rod/poly(1,10-phenanthroline-5,6-dione)/gold nanoparticles/poly(pyrrole-2-carboxylic acid)) electrode was modified by glucose oxidase (Figure 4a) [58]. The PPCA was used to encapsulate the AuNPs in the prepared biosensor. The GOx covalent immobilization was conducted via the activation of carboxyl groups located in the polymer PPCA with a mixture composed of 200 µL of 0.4 M EDC and 200 µL of 0.1 M NHS. The reaction between PPCA, EDC and NHS resulted in a semi-stable NHS ester which then reacts with the amine groups of the enzyme. The influence of pH of the GOx solution on the analytical signal was investigated, and the highest current was observed for pH 4 (Figure 4b). Different amounts of GOx were also tested in order to find the best one (Figure 4b). The increase in GOx concentration resulted in the growth of the current, and finally, 40 mg/mL was chosen as the most appropriate amount. Furthermore, the activation time of EDC/NHS, as well as GOx, was optimized by immersing the electrodes in the solutions for 10, 20, 30 and 40 min (Figure 4b). The 30-minute duration was concluded to be adequate to perform efficient modification. The relationship between the current and glucose concentration was investigated, and the linear relationship from 0.20 mM to 150.00 mM was observed (Figure 4c). These results have confirmed that the electrode could be used for glucose detection in samples containing a large amount of that sugar.

### 3.2. Adsorption

Enzyme immobilization by adsorption is the most direct method for enabling the integration of the biological molecule with the substrate. The mechanism is based on the creation of weak bonds such as hydrophobic and electrostatic interactions or Van der Waal’s forces [52]. The enzyme is dissolved in solution which is most often a phosphate buffer and subsequently, the solution is dropped on the electrode, or the samples are simply immersed in it. Then, the unabsorbed molecules should be removed from the surface by washing with buffer. This method is one of the easiest and cheapest ones since there are no additional ingredients used for the functionalization, and the whole procedure is not complicated.

**Figure 5:**
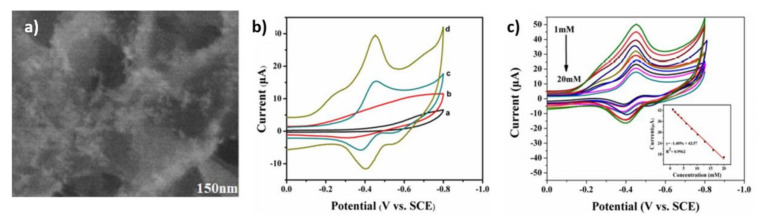
Image (**a**), SEM image of Au-functionalized 3D hierarchically ZnO; image (**b**), the cyclic voltammetry curves registered for (**a**) ZnO/GCE, (**b**) Au-ZnO/GCE, (**c**) GOx/ZnO/GCE, (**d**) GOx/Au-ZnO/GCE in N_2_ saturated phosphate buffer solution; image (**c**), in O_2_ saturated PBS in various concentration of glucose for the GOx/Au-ZnO/GCE. Reprinted with permission from [59]; Copyright 2016, Elsevier.

The AuNPs-functionalized 3D hierarchically ZnO material (Figure 5a) was modified using the adsorption immobilization method [59]. The 8 µL of GOx solution was dropped onto the AuNPs-ZnO/GCE electrode and then dried at ambient temperature. The CV measurements (Figure 5b) for various configurations of electrodes were presented. It should be noted that the redox peaks were observed for two electrodes containing glucose oxidase, such as GOx/ZnO/GCE and GOx/AuNPs-ZnO/GCE, where the anodic peak is attributed to the oxidation of FADH_2_ (reduced flavin adenine dinucleotide) and the cathodic peak to the reduction in FAD (flavin adenine dinucleotide). It should be mentioned that an electrode with gold nanoparticles is the most favorable one, and this electrode was selected for further examination (Figure 5c). The cathodic peak which is decreasing due to successive glucose addition was analyzed. It was established that the linear range of the glucose concentration is 1.00–20.00 mM, the sensitivity is equal to 19.85 µA mM^−1^ cm^−2^, while the limit of detection is 0.02 mM.

**Figure 6:**
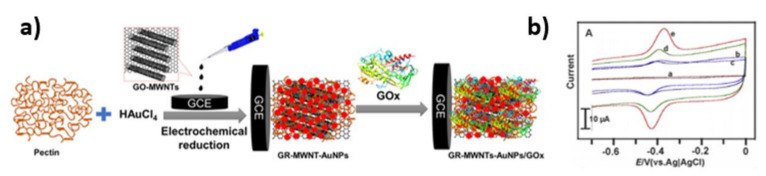
(**a**) Scheme of the fabrication of GCE modified with a GR-MWNTs/AuNPs/GOx layer, (**b**) cyclic voltammograms obtained for the modified electrodes. Reprinted with permission from [60]; Copyright 2015, Elsevier.

The glucose sensor based on the GR-MWNTs/AuNPs/GOx electrode was developed by Devasenathipathy et al. [60] The sample was composed of a glassy carbon electrode modified by graphene (GR) multiwalled carbon nanotubes (MWNTs) with pectin stabilized gold nanoparticles and glucose oxidase. The enzyme immobilization was performed by dropping 5 µL GOx onto the GR-MWNTs/AuNPs film which was then dried at room temperature. A schematic representation of the fabrication process of the electrode is shown in Figure 6a. The cyclic voltammograms of: (a) AuNPs/GOx, (b) MWNTs/AuNPs/GOx, (c) GR/AuNPs/GOx, (d) GR-MWNTs/GOx and (e) GR-MWNTs/AuNPs/GOx in phosphate buffer are presented in Figure 6b. The redox peaks of GOx at ca. −0.4 V can be observed for all electrodes except the AuNPs/GOx. The GR-MWNTs/AuNPs/GOx exhibits the most effective electron transfer, recognized as the highest peak currents, and this activity results from the developed surface area and conductivity.

**Figure 7:**
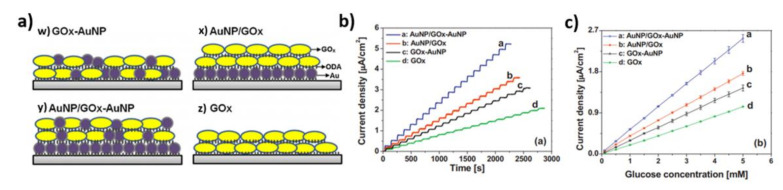
(**a**) Schematic representation of Langmuir–Blodgett films; (**b**) amperometric responses measured for different types of electrodes; (**c**) the corresponding calibration curves. Reprinted with permission from [61]; Copyright 2016, Elsevier.

The Langmuir–Blodgett (LB) deposition technique is an extension of the adsorption method of enzyme immobilization. The LB method can be used to immobilize enzymes by co-adsorption of molecules and nanoparticles on the electrode surface [61]. The Langmuir–Blodgett technique allows for the control of both the architecture and film thickness. Following Ke-Hsuan Wang et al., the glucose oxidase and gold nanoparticles were prepared for the glucose-sensing system. The first sample (Figure 7a(w)) was composed of two layers of GOx-AuNP mixed monolayer on Pt. Another examined electrode was the AuNP/GOx (Figure 7a(x)) which included a closely packed AuNP film on Pt and two layers of GOx. The third electrode also had a gold layer and two mixed monolayers of GOx-AuNP (Figure 7a(y)). The last electrode was composed of two monolayers of GOx placed directly on Pt, and it was used as the reference (Figure 7a(z)). In each case, an octadecylamine (ODA) was used as a co-adsorbent. The CA measurement performed at +0.6 V (vs. Ag/AgCl) in 0.1 M PBS was used to evaluate glucose sensing. As is seen in Figure 7b, the current increased after each injection of glucose. On the basis of these results, calibration curves were established (Figure 7c). Among the four electrodes, the single GOx film showed the lowest sensitivity, equal to 0.21 µA mM^−1^ cm^−2^. The GOx-AuNP and the AuNP/GOx electrodes exhibited a sensitivity of 0.31 µA mM^−1^ cm^−2^ and 0.36 µA mM^−1^ cm^−2^, respectively. The best result, defined here as the highest sensitivity of 0.52 µA mM^−1^ cm^−2^, was found in the AuNP/GOx-AuNP sample. The composition of electrodes had a distinct effect on the sensitivity enhancement, in terms of glucose sensing.

### 3.3. Cross-Linking

The enzyme immobilization with the use of the cross-linking technique requires the formation of the connection between enzyme molecules by linkers. Such an approach leads to the creation of three-dimensional cross-linked aggregates [52]. The high interaction strength causes low enzyme leakage and an increase in enzymatic stabilization. However, the agents used for the immobilization, as well as the process conditions, can cause many stresses in the enzyme molecule, leading to protein modification or even loss of enzymatic activity [55]. Moreover, the reagents used for the modification are often expensive and toxic.

**Figure 8:**
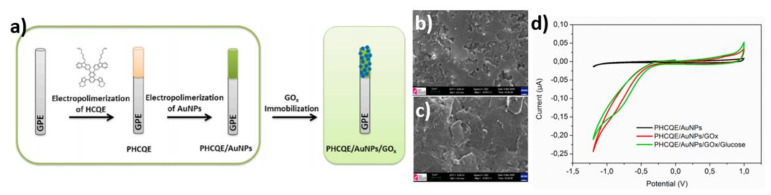
(**a**) Schematic representation of the preparation of PHCQE/AuNPs/GOx;(**b**) SEM image of PHCQE/AuNPs; (**c**) SEM image of PHCQE/AuNPs/GOx; (**d**) CV curves for PHCQE/AuNPs and PHCQE/AuNPs/GOx electrodes in 0.1 M PBS without and with glucose. Reprinted with permission from [62]; Copyright 2020, Elsevier.

The cross-linking method was used for GOx immobilization on the PHCQE/AuNPs electrode [62]. Firstly, the HCQE (3-(5,8-bis (2,3-dihydrothieno[3,4-b][1,4]dioxin-5-yl)-3-(9-hexyl-9H-carbazole-3-yl) quinoxalin-2-yl)-9-hexyl9H-carbazole) was electropolymerized on to a GPE (glass pencil electrode). Secondly, the HCQE was enriched by AuNPs. The enzyme immobilization was performed by immersing a sample in glucose oxidase and glutaraldehyde solution for 30 min at 8 °C and then dried for another 30 min (Figure 8a). Figure 8b shows the surface of PHCQE/AuNPs, on which gold nanoparticles are unevenly distributed, in contrast to the morphology given in Figure 8c where nanoparticles underwent enzymatic functionalization. The electrochemical activity for the PHCQE/AuNPs and the PHCQE/AuNPs/GOx electrodes was characterized using cyclic voltammetry carried out in 0.1 M PBS solution (Figure 8d). It can be observed that different shapes of CV curves were achieved for those electrodes, caused by the presence of GOx on one of them. Those differences can be identified by comparing currents recorded at −1.2 V and +1.0 V. For the PHCQU/AuNPs an electrode current of −0.025 µA at −1.2 V was achieved, while for the PHCQU/AuNPs/GOx that value was 10 times higher. In addition, the visible oxidation reaction at ca. +0.8 V and the reduction reaction at ca. −0.8 V can be observed for the enzymatic sample, in contrast to the non-enzymatic one. The obtained result confirms the activity toward glucose for polymer-based nanoparticles modified enzymatic electrodes. The most prominent reduction peak caused by the glucose addition is located at ca. −0.7 V. The PHCQU/AuNPs/GOx biosensor exhibited a linear response from 0.75 to 3.13 mM, a sensitivity of 0.13 µA mM^−1^ and the LOD reaching 0.02 mM.

**Figure 9:**
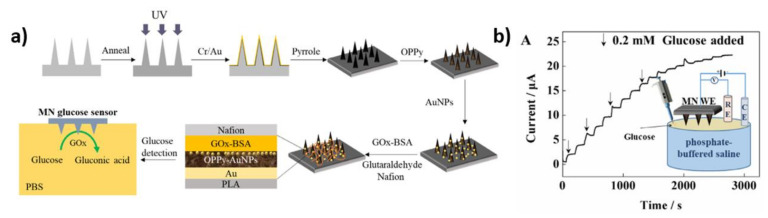
(**a**) Fabrication process of the Nafion/GOx/AuNPs/OPPy/AuMNs electrode, (**b**) chronoamperometry curve of the electrode in PBS with the successive addition of glucose. Reprinted with permission from [63]; Copyright 2020, Elsevier.

Another example is an electrode composed of polylactic acid (PLA) microneedle (MN) coated with gold, over oxidized polypyrrole (OPPy), gold nanoparticles, enzyme and Nafion [63]. A schematic representation of the preparation of samples is shown in Figure 9a. At the beginning of the immobilization process, the solution of the glucose oxidase (25 mg/mL) and the bovine serum albumin (BSA) (10 mg/mL) were fixed in a PBS solution, then 10 µL was dropped onto the electrode surface and dried at 4 °C for 6 h. Subsequently, 10 µL of 2% GA solution was deposited. Finally, the electrodes were covered with a Nafion layer. The successive addition of 0.2 mM of glucose resulted in a gradual increase in the current values during the CA measurement at +0.75 V (Figure 9b). Furthermore, the following sensor parameters for the GOx/AuNPs/OPP/AuMNs electrode were determined: sensitivity—8.09 μA mM^−1^, limit of detection—40.00 μM, linear range—from 0 to 2.60 mM. For comparison, a lower sensitivity of c.a. 3.11 μA mM^−1^ for the GOx/AuMNs sample without AuNPs and OPPy was obtained. The results proved that the addition of gold nanoparticles and overoxidized polypyrrole increases the catalytic area of fabricated samples.

In comparison to previous approaches, based on enzyme immobilizations by cross-linking, used for the fabrication of PHCQU/AuNPs/GOx and GOx/AuNPs/OPP/AuMNs, in this case, the glucose oxidase was applied repeatedly. The platform for the enzyme was graphite rods (GR) covered by Fe_3_O_4_-CS-Au magnetic nanoparticles [64]. The iron nanoparticles were first covered by chitosan and then immersed in HAuCl_4_ solution in order to create gold-covered iron oxide nanoparticles. The glucose oxidase solution was dropped onto the Fe_3_O_4_-CS-Au/GR electrode three times. Such a procedure was proposed in order to improve sensor performance and immobilize more of the GOx on the available surface. Subsequently, the modified sample was stored in a 1% solution of glutaraldehyde for 24 h. Among all the tested electrodes, the highest density current after 5 mM glucose addition to the solution was reached by the Fe_3_O_4_-CS-Au-GOx composition. The value was about 2 times higher than for the Fe_3_O_4_-CS-GOx and 4 times higher compared to the Fe_3_O_4_-GOx. It was concluded that the combination of the Fe_3_O_4_-CS-Au provided a higher signal intensity as well as stability of the immobilized enzyme than electrodes without gold. The chronoamperometry measurement for the best electrode showed a linear increase in current after glucose injections. The LOD was determined as 0.55 mM and the range of linear response was from 5.00 to 30.00 mM.

### 3.4. Entrapment

The entrapment method is based on the trapping of the enzyme in a polymeric matrix, allowing the transport of products and substrates of the reaction, but enabling the hosting of the enzyme inside the matrix [54]. The advantage of this method is the preservation of enzyme activity, and usually, this approach does not affect enzymatic structure [65]. The enzyme is not directly bonded to the electrode surface but is simply trapped in a polymer. However, in the case of non-optimized polymer concentration, a limitation in mass transport may occur.

**Figure 10:**
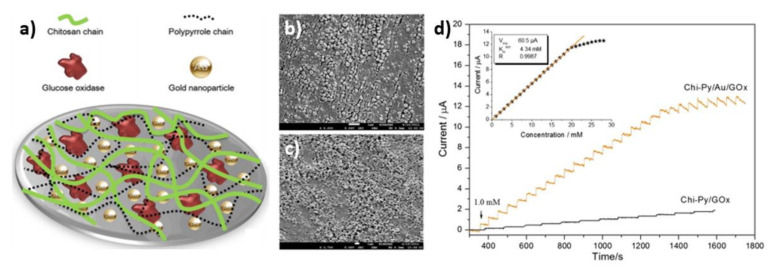
(**a**) Schematic structure of glucose sensor electrode; (**b**) SEM image of the Chi-Py/Au; (**c**) SEM image of the Chi-Py/Au/GOx; (**d**) amperometric response of the enzymatic electrodes. Reprinted with permission from [66]; Copyright 2015, Elsevier.

Acting according to Senel [66], the enzyme was entrapped in the GCE/Chi-Py/Au/GOx material. The fabrication of the enzymatic electrode was conducted in a few steps. Firstly, the pyrrole-branched-chitosan (Chi-Py) was prepared. The chitosan was dissolved in an acetic acid solution and then diluted in methanol. Secondly, the pyrrole (PyPA) and the EDC were added, and all ingredients were stirred together. After 24 h the mixture was pureed and prepared for the next stage of the process. Finally, the connection between Chi-Py, GOx and HAuCl_4_ was established, and that mixture was deposited onto the GCE surface (Figure 10a). The SEM images of the surfaces of Chi-Py/Au and Chi-Py/Au/GOx are shown in Figure 10b,c, respectively. As can be seen, uniform gradual morphology with Au nanoparticles was obtained in the Chi-Py/Au sample (Figure 10b). When the GOx was immobilized on the material the morphology was changed, so a regular and porous structure was formed (Figure 10c). The electrochemical activity of GCE, GCE/Chi-Py and GCE/Chi-Py/Au samples was tested in 10 mM PBS solution containing [Fe(CN)_6_]^3−/4−^ in order to choose the best platform for enzyme entrapment. The highest current response in the presence of the redox couple was observed for the GCE/Chi-Py/Au electrode. Subsequently, the GOx was immobilized on GCE/Chi-Py and GCE/Chi-Py/Au, and the amperometric response during the successive addition of glucose was investigated (Figure 10d). The electrode containing gold nanoparticles showed a four-times higher response than for one without Au. For the best electrode, the linear range from 1.00 to 20.00 mM and the sensitivity of 0.58 μA mM^−1^ were determined.

**Figure 11:**
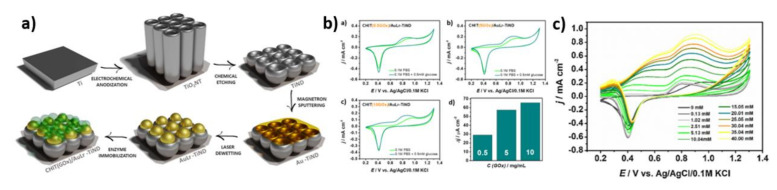
(**a**) The diagram showing the preparation of enzyme-modified electrodes; (**b**) CVs curves registered for different glucose oxidase concentrations; (**c**) CVs curves for the CHIT(GOx)/AuLr-TiND with glucose addition. Reprinted with permission from [28]; Copyright 2021, Elsevier.

Another example of the application of entrapment modification was the fabrication of the CHIT(GOx)/AuLr-TiND electrode [28]. The glucose oxidase was immobilized by entrapment in a chitosan matrix on a nanostructured titanium foil with laser-produced gold nanoparticles. The schematic fabrication process is shown in Figure 11a. As can be seen in Figure 11b, the current value at +0.8 V vs. Ag/AgCl/0.1 M KCl increases with the growing amount of enzyme concentration; however, between 5 and 10 mg/mL GOx, only a 10% difference in the electrochemical response is observed, and it is the reason why a plateau was achieved. Based on the cyclic voltammetry measurements (Figure 11b), two linear ranges were distinguished at 0.04–15.05 mM and 15.05–40 mM, the sensitivity was estimated as 23.47 ± 1.36 and 10.63 ± 1.28 μA mM^−1^ cm^−2^, respectively, and the limit of detection was concluded to be 1.75 ± 0.30 μM. In order to determine the effect of enzymes on the sensor performance, a comparison with a non-modified electrode has been made. The CHIT(GOx)/AuLr-TiND electrode has a lower limit of detection than the non-enzymatic one (8.4 μM), as well as a wider linear range than ldTiND/AuNPs (0.01–1 mM) [67]. However, the enzymatic modification reduces the sensitivity.

**Figure 12:**
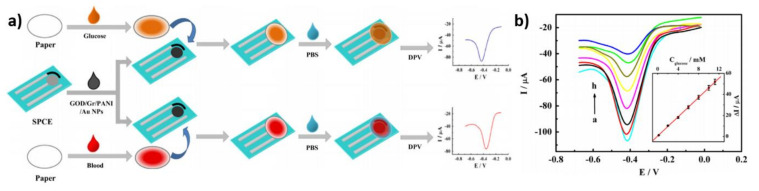
(**a**) Schematic representation of the preparation of Gr/PANI/AuNPs/GOD/SPCE electrode, (**b**) DPV curves of modified SPC electrode in 0.1 M PBS with glucose addition. Reprinted with permission from [68]; Copyright 2014, Elsevier.

Taking into account the commercial applications, one should also consider screen-printing (SP) technology for electrode material preparation, including anchoring of the enzyme molecule. Acting according to Kong et al. [68], the glucose oxidase was entrapped in a polymer matrix and immobilized on an SP electrode. It was composed of graphene (Gr), polyaniline (PANI), gold nanoparticles, glucose oxidase (GOD), chitosan (CS), a screen-printed carbon electrode (SPCE) and Nafion. The schematic representation of the fabrication process of the electrode is shown in Figure 12a. Firstly, the graphene/polyaniline/gold nanoparticles nanocomposite was added to a 0.5% chitosan solution. Secondly, the glucose oxidase solution was mixed with Gr/PANI/AuNPs/CS suspension and then shaken for 30 min. Next, 8 µL of the mixture was dropped on the SPCE surface. Finally, the electrode was covered by Nafion acting as a protective, semipermeable membrane. The electrochemical activity of the modified SPCE electrode was tested in 0.1 M PBS with subsequent glucose addition (Figure 12b). The limit of detection and sensitivity was equal to 1.0 × 10^−^^4^ mol/L and 20.32 µA cm^−^^2^ mM^−^^1^, respectively. The advantage of the SPCE electrode is the possibility of determining glucose in a very low volume of the tested probe—only 2 µL is required.

### 3.5. Self-Assembled Monolayers (SAMs)

SAMs are self-assembled monolayers of organic compounds which are formed on the surface by chemisorption [69]. SAMs are composed of three parts. The first one—the head group—is the end of the molecule containing a functional group such as thiol or disulfide, which is connected to the platform. The second one—the backbone—is an aliphatic chain or an aromatic oligomer. This part is responsible for molecular ordering. Finally, the third one, known as the terminal group, is accountable for the chemistry of the constructed layer and makes it possible for the enzyme to be attached [70]. It should be mentioned that this method is usually characterized by numerous difficult procedures of modification, as well as expensive reagents.

The most commonly used SAMs-modified materials for glucose biosensor application are unpatterned gold electrodes. Acting according to Shervedami et al., the polycrystalline gold working electrode was modified by a water/ethanol solution of 20 mM MPA (3-mercaptopropionic acid), in which it was placed for 12 h, and then activated by 5 mM NHS and 0.2 mM EDC. Next, the GOx was immobilized for 1.5 h to fabricate the Au-MPA-GOx SAMs electrode [71]. The prepared electrode was used to determine the glucose content in 0.1 M PBS solution with the addition of parabenzopinone (PBQ) mediator by EIS (electrochemical impedance spectroscopy). The linear relation of 1/R_ct_ and glucose concentration was found to be within the range of 0–10.00 mM.

Acting according to Zhong et al. [72], the material was composed of two silane layers of a 2d-network of (3 mercaptopropyl)-trimethoxysilane MPS and self-assembled gold nanoparticles, as well as an enzyme. The gold electrode was immersed in an ethanol solution of 40 mM MPS for 3 h. Next, the electrode was immersed in a 0.01 M NaOH solution for two hours in order to form a hydrolyzed and condensed monolayer. In another step, the second layer was created by dipping it back in MPS overnight. After that, the sample was dipped in the AuNPs solution for 10 h. In the end, the GOx enzyme was immobilized at 4 °C overnight. The addition of the second layer of MPS resulted in an increase in the surface area as well as an increase in enzyme loading. As a result, higher sensitivity and stability were achieved.

**Figure 13:**
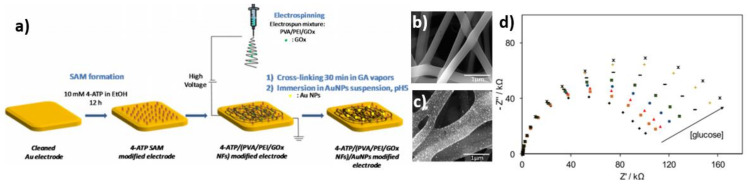
(**a**) Scheme of the fabrication process of the biosensor; (**b**) SEM image of PVA/PEI NFs; (**c**) SEM image of PVA/PEI NFs/AuNPs; (**d**) Nyquist plots of impedance spectra for the ATP/PVA/PEI/AuNPs/GOx electrode with glucose addition in phosphate buffer solution. Reprinted with permission from [73]; Copyright 2017, Elsevier.

Another example of a glucose biosensor in which SAMs were used is enzymatic electrospun nanofibers decorated with AuNPs [73]. The process of electrode fabrication is shown in Figure 13a. Firstly, the gold electrode was incubated in an ethanol solution of 10 mM 4-ATP (4-aminothiophenol) for 12 h for the formation of the 4-ATP SAM modified electrode. Secondly, the electrospinning process and the preparation of an electrospun solution took place. The mixture was composed of PVA (poly(vinyl alcohol)), PEI (poly(ethyleneimine)) and a glucose oxidase. The concentration of polymer was equal to 12 wt% with a mass ratio of 3:1 PVA/PEI and 15 mg/mL GOx per mL of a polymer. The nanofibers were directly formed on the surface of the Au electrode during electrospinning. Thirdly, the process of NFs cross-linking by glutaraldehyde vapors was initiated. In the end, the electrode was immersed in the AuNPs solution. The SEM images of the PVA/PEI NFs and PVA/PEI NFs/AuNPs surfaces are shown in Figure 13b,c. As can be seen in Figure 13d, the impedance increases with glucose addition in the range of 0–1.00 mM, and is caused by the accumulation of reaction products at the electrode surface. For the 4-ATP/PVA/PEI/AuNPs electrode without an enzyme, no significant response was obtained. It is possible that the abovementioned modification combines two immobilization methods—not only SAMs but also the entrapment of GOx in the polymer matrix.

The comparison of the performance of various electrodes modified using different types of immobilization methods is shown in Table 2. The case studies were carried out using cyclic voltammetry, chronoamperometry and electrochemical impedance spectroscopy techniques. In order to compare results, sensor parameters such as sensitivity, linear range and detection limit were listed. The lowest limit of detection equals 0.1 nM and was achieved for the GOD/Fc/Au/SLG/GCE electrode modified using a covalent linking method, and the 2dMPS-AuNPs-GOx electrode in which SAMs was used for enzyme immobilization. The highest sensitivity, accompanied by a wide linear range, was reached by the electrode fabricated via the covalent bonding method—M3(GOx)/Au-TiND (25.74 µA cm^−2^ mM^−1^) and entrapment method—CHIT(GOx)/AuLr-TiND (23.47 µA cm^−2^ mM^−1^).

## 4. Conclusions

The different enzyme immobilization methods with AuNPs for glucose sensing were described in this paper. The most commonly used techniques for gold nanoparticle preparation have been also introduced. However, special attention was put on covalent bonding, adsorption, cross-linking, entrapment and self-assembled monolayers, which are recognized as the most frequently applied methods for enzyme immobilization. Electrodes modified using covalent linking and self-assembled monolayer techniques achieved the lowest limit of detection among all types of immobilization. This could be the result of ordered molecular arrangements and fast electron transfer along the chains. At the same time, the highest sensitivity and the widest range of linear response were obtained when cross-linking and entrapment techniques were used. In this case, the number of enzymes immobilized on the electrode surface and the thickness of the functionalization layer can be greater than for adsorption, covalent bonding and SAMs modifications. As a result of the utilization of highly advanced methods of nanomaterials fabrication and novel characterization techniques [74] as well as progress in theoretical and computational methods [75], the inevitable and fast development of enzyme immobilization and sensing systems is expected. The multianalyte detection of glucose, lactose, fructose, cortisol, dopamine, vitamin C or paracetamol, and more, will be expanded [76]. We believe that the development of protective biofouling materials [77], as well as the minimization of sensors and wearable technology [78], will receive great attention. The huge number of articles as well as projects concerning sensors, among others glucose biosensors, lead us to the conclusion that they will become an integral part of human life. We also strongly believe that sensors will contribute particularly to the development of rapid medical diagnostics. Of course, one cannot forget that the way to transfer the proof-of-concept devices that are described in the literature to real-life applications should occur first, as it still remains the most critical issue to be solved. In such a case scenario, electrochemical sensors will be used to their full potential. We are confident that in the near future it will be possible for each individual to be able to examine the analytes in his/her body at the most convenient time. Moreover, the obtained results will be simultaneously sent to the doctor, enabling the fast implementation of treatment. Collecting health data, fast analysis and medical advice will become a daily reality.

## Figures and Tables

**Table 1:** The comparison of non-enzymatic electrodes containing AuNPs for glucose detection in alkaline solution, as reported in the literature.

Electrode	AuNPs Support Material	AuNPs Fabrication Method	Sensitivity (µA cm^−2^ mM^−1^)	Linear Range (mM)	Detection Limit (µM)	Ref.
GO-COO Au/GCE	carboxylated graphene oxide/glassy carbon electrode (GCE)	HAuCl_4_ chemical reduction	20.20	0.02–4.58	6.00	[43]
Au/LSGEs	laser-scribed graphene	electrodeposition	-	0.01–10.00	6.30	[44]
Au/PPyNFs	polypyrrole nanofibers/GCE	HAuCl_4_ chemical reduction	1.003	0.20–13.00	-	[45]
AuNPs/CuO NWs	copper oxide nanowires/Cu	HAuCl_4_ chemical reduction	4398.80	0.0005–5.90	0.50	[46]
AuNPs/ZnO NRs	zinc oxide nanorods/ITO	HAuCl_4_ chemical reduction	157.30	0.50–10.00	0.06 mM	[47]
AuNPs/ITO	indium tin oxide	electrodeposition	23.00	up to 11.00	5.00	[48]
GNP/MWNT CR	multi-walled carbon nanotubes/Au	electrodeposition	-	up to 5.00	0.50	[49]
Au/ITO	indium tin oxide	ion implantation	-	0.001–0.17 and0.20–15.00	0.40	[50]
AuNPs/TiO_2_NRs/FTO	fluorine tin oxide (FTO)	thermal evaporation	0.01	0–10.00 and 10.00–30.00	-	[51]

**Table 2:** The comparison of the performance of electrodes reported in the literature for different types of immobilization method (IM).

Electrode	IM	Sensitivity (µA cm^−2^ mM^−1^)	Linear Range (mM)	Detection Limit (mM)	Ref.
Graphene-GOx-GNP	CB	-	-	0.03 mg/mL	[56]
GOD/Fc/Au/SLG/GCE	CB	-	0.10 nM–5.00	0.10 nM	[57]
GR/PPD/(AuNP)PPCA-GOx	CB	0.14 µAmM^−1^	0.20–150.00	0.08	[58]
GOx/Au-ZnO/GCE	A	19.85	1.00–20.00	0.02	[59]
GR-MWNTs/AuNPs/GOx	A	0.70	10.00 µM–2.00	4.10 µM	[60]
AuNP/GOx-AuNP	A	0.52	0.10–5.00	63.00 µM	[61]
PHCQU/AuNPs/GOx	CL	0.13 µAmM^−1^	0.75–3.13	0.02	[62]
Nafion/GOx/AuNPs/OPPy/AuMNs	CL	8.09 μA/mM	0–2.60	40.00 μM	[63]
Fe_3_O_4_-CS-Au-GOx	CL	-	5.00–30.00	0.55	[64]
M3(GOx)/Au-TiND	CL	25.74	0.05–3.05	7.61 μM	[27]
GCE/Chi-Py/Au/GOx	E	0.58 μA/mM	1.00–20.00	-	[66]
CHIT(GOx)/AuLr-TiND	E	23.47 10.63	0.04–15.05 15.05–40.00	1.75 μM	[28]
Au-MPA-GOx SAMs	S	-	0–10.00	-	[71]
2dMPS-AuNPs-GOx	S	-	0.40–52.80 nM	0.10 nM	[72]
ATP/PVA/PEI/AuNPs/GOx	S	-	0–1.00	-	[73]

CB—Covalent bonding, A—Adsorption, CL—Cross-linking, E—Entrapment, S—Self-assembled monolayers.

## Abbreviations

AuNPs	gold nanoparticles
AACVD	aerosol-assisted chemical vapor deposition
BSA	bovine serum albumin
CVD	chemical vapor deposition
CV	cyclic voltammetry
CA	chronoamperometry
CS	chitosan
CHIT	chitosan
–CHO	aldehyde group
Chi-Py	pyrrole-branched-chitosan
DPV	differential pulse voltammetry
EDC	N-ethyl-N’-(3-dimethylaminopropyl)carbodiimide hydrochloride
EIS	electrochemical impedance spectroscopy
FADH_2_	reduced flavin adenine dinucleotide
FAD	flavin adenine dinucleotide
Fc	6-(ferrocenyl)hexanethiol
GNPs	gold nanoparticles
GOx	glucose oxidase
GOD	glucose oxidase
GCE	glassy carbon electrode
GR	graphene rod
HCQE	3-(5,8-bis (2,3-dihydrothieno[3,4-b][1,4]dioxin-5-yl) -3-(9-hexyl-9H-carbazole-3-yl) quinoxalin-2-yl);
HAuCl_4_	gold (I) perchlorate
ITO	indium tin oxide
IDE	interdigitated (di)electrodes
LOD	limit of detection
LB	Langmuir–Blodgett method
MPA	3-mercaptopropionic acid
MUA	mercaptoundecanoic acid
MN	microneedle
MWNTs	multiwalled nanotubes
MPS	(3 mercaptopropyl)-trimethoxysilane
NHS	N-hydroxysuccinimide
–NH_2_	amino group
ODA	octadecylamine
OPPy	oxidized polypyrrole
PVD	physical vapor deposition
PPD	poly(1,10-phenanthroline-5,6-dione)
PPCA	poly(pyrrole-2-carboxylic acid)
PCE	pencil carbon electrode
PLA	polylactic acid
PVA	poly(vinyl alcohol)
PEI	poly(ethyleneimine)
PBQ	parabenzopinone
–SH	thiol group
SLG	single-layer graphene
SEM	scanning electron microscopy
SAMs	self-assembled monolayers
SP	screen-printing
SPCE	screen-printed carbon electrode
TiND	titanium nanodimples
4-ATP	4-aminothiophenol
